# Simplified 3D hydrodynamic flow focusing for lab-on-chip single particle study

**DOI:** 10.1038/s41598-023-40430-z

**Published:** 2023-09-06

**Authors:** Filippo Storti, Silvio Bonfadini, Luigino Criante

**Affiliations:** 1 Center for Nano Science and Technology, Istituto Italiano di Tecnologia, Via Rubattino 81, 20134 Milano, Italy; 2 Department of Physics, Politecnico di Milano, Piazza Leonardo da Vinci, 32, 20133 Milano, Italy

**Keywords:** Optofluidics, Fluid dynamics, Design, synthesis and processing, Laser material processing

## Abstract

Accurately control of the position of a fluid and particle within lab-on-a-chip platform is a critical prerequisite for many downstream analysis processes, such as detection, trapping and separation, moving the sensing at the single-particle level. With the development of microfluidic fabrication technology, particle/cell focusing has shifted from two to three dimensions. 3D hydrodynamic focusing, which sorts and aligns the incoming cloud of particles so that they pass through the interrogation area one by one, enables new possibilities and breakthroughs in the single-cell analysis system. Despite the excellent results shown in literature, there is still a lack of a device that can simultaneously fulfilling the requirements of high throughput, compactness, high integrability, and ease of use operation to become a widely accepted work center for biomedical research and clinical applications. Here, we proposed a unique 3D flow focusing microfluidic device buried in fused silica substrate that potentially combines all this advantages. By designing a sample channel suspended inside a larger buffer channel, manufactured by exploiting the laser-assisted micromachine technique, a not size-dependent focusing capability is shown. A spatially and temporally stable central flow of a mixture of 15 μm and 6 μm PS particles to a 1 μm PS microsphere solution has been obtained with high accuracy. Finally, to test the achievable focusing resolution, the chip was tested for the detection of Escherichia Coli bacteria in water solution as proof of concept of biological application.

## Introduction

The precise manipulation of fluids on board of microfluidic platforms has aroused in the recent years the attention of the Lab-On-a-Chip (LOC) research field, due to the wide range of application that can be implemented. Besides drastically reducing the amount of processing samples and shrinking the size of the instruments to a portable scale, the capability to accurately control the position of a fluid—thus, of the particles flowing in it as well—within microfluidic devices has shifted the sensing at the single particle level. This greatly improves the sensitivity and the amount of extractable information. Multiple fields have already benefited of these advantages such as the bio-medical one—e.g., flow cytometry and single particle detection^1^—or the environmental one—e.g., climate change monitoring, microbial water contamination^2^—and industry—cosmetics^3^, and food and beverage purity control^2^. Particles flowing within a microfluidic platform, especially at high concentrations, distribute randomly in the cross-section of the channel, drastically affecting the efficiency of any further analysis step^4^. Thus, the main principle behind 3D flow focusing is ordering the incoming cloud of particles, aligning them so that they cross the interrogation area one by one. Therefore, from this perspective, the ideal focusing device should take in account the presence of one or more downstream analysis steps (e.g. detection, trapping and separation), while to be able to manage very small particle and even molecules, a not size-dependent focusing capability is a game-changer. For this reason, critical requirements to be fulfilled are compactness, to not affect the portability of the platform, high throughput, not to slow down the whole analysis process, and ease-of-use, to make the device as automated and flexible as possible. Many different strategies have already tried to face this challenge. Mainly, two distinct approaches can be identified: methods that induce forces on particles externally (a.k.a. active) or internally (a.k.a. passive). In the first case, the most used force fields are acoustic^5, 6^, magnetic^7^ and electric^8, 9^. Although they are effective methods, the need of another external stimuli complicates both the fabrication process, needing the integration of elements such as piezo transducers, magnets, and electrodes, and the operation, requiring the control of the force generation in addition to the flow. Then, to let the field act on the particle trajectory, the flow rates need to be limited, resulting in throughputs from 0.85 µL/min to few hundreds of μL/min and just in limited cases around 500 µL/min^4, 6^. On the other hand, passive methods exert a focusing action just thanks to their flow configuration. In this case another distinction can be made: some approaches use sheath-less effects, due to inertial forces generated by the channel geometry^10^ or by the integration of structures, such as grooves or pillars, in the channel^11, 12^, while others use multiple inlets, from 1 to 4, to confine the sample flow^13–16^. Inertial microfluidics is an attractive method since allows to achieve high throughputs (up to few mL/min)^17^ and no sheath flows are needed, thus reducing the operational complexity. However, inertial focusing has difficulty in improving the focusing resolution especially in small space. In fact, it is well known that these devices exploit the competition between two forces acting on the particles flowing, that are strongly related to their size: the shear-induced lift forces and the wall-induced lift forces^17^. Thus, to achieve flow focusing it is necessary to use just one particle diameter at a time. Furthermore, focusing particles close to the (sub-)micrometer scale encounters difficulties, as the minimum microchannel length required for effective focusing increases dramatically as particle size decreases, affecting the chip compactness and complicating the integration of further analysis steps. In straight channels, the geometry and the focusing length vary depending on the particle diameter^10, 18–21^, while in the case of spiral channels^22, 23^ or contraction–expansion arrays^24^, by using a mixture of different sizes, different equilibrium points are set at different positions across the channel section. This results in multiple focused flows which are unsuitable for a single interrogation region. Then, inertial effects are seen when the volume fraction of particles is less than 1%—to avoid that particle–particle interactions disrupt the focusing^25^. This means that the sample must be diluted, decreasing the effective throughput, and requiring an additional pre-processing step. The conceptually easiest way to confine the sample flow at the center of a microfluidic channel, regardless of particle size, is to inject other four flows within the same channel. Many works have implemented such strategy, achieving good focusing results^14, 16, 26, 27^, but still the need for managing five (or six) inlets simultaneously does not facilitate the device's use in clinical applications. For this reason, several groups have presented intriguing solutions to reduce the number of injection ports. One of the most used strategies is to split an original branch before connecting it to the main channel, decreasing the number of inlets at two^13, 28, 29^. Besides the reduced complexity in the operation of such devices, the flow must be precisely partitioned in all the branches to reach an accurate particle positioning. Thus, the risk of introducing asymmetries in the fluid manipulation or in the geometry, during the fabrication processes or in the experimental functioning—e.g., due to an air bubble—increases. Tripathi et al.^30^ have achieved a simplified geometry with just two inlets and no splitting, which exploits the combination of a buffer flow and the Dean vortex effect due to curved channels. Besides the good focusing efficiency, the throughputs are limited at 100 µL/min. Instead, some other works have reached trade-off by using two sheath inlets and reducing the aspect ratio of the sample channel, so that the buffer flow envelops the main flow at the junction^31, 32^. Nonetheless, also these devices show a limited throughput ranging from µL/min to 30 µL/min, mainly due to the de-formability of polydimethylsiloxane (PDMS), of which they are made. Instead, Patel et al.^33^ have used a similar strategy, but their device flow rate is not limited thanks to the polymethyl methacrylate (PMMA) micromilling. However, since their focused particles flow on the bottom of the main channel, the device is exposed to clogging risks. Other creative solutions are represented by attempts of surrounding a main channel by buffering inlets, by integrating two micropipettes^34^, several micro-capillaries^35^, or a micro-nozzle^36^. Besides the elegant implementation, the devices are very fragile, and the performances are strictly linked to the accuracy of the fabrication process.

In this work we present a microfluidic network for the precise positioning of particles at the center of the main channel, exploiting only two inlet (sample and sheath flow), that aims at fulfilling the requirements—i.e., ease-of-use, high throughputs, and compactness—to push forward the platform integrability. The device design is studied throughout numerical simulations and then fabricated buried in fused silica substrate thanks to the high versatility of Femtosecond Laser Irradiation, followed by Chemical Etching (FLICE) technique^37–41^. Besides its great 3D capabilities, this well-known technique also offers the possibility to integrate other analysis elements—such as optical elements^42^, optical fibers, and electrodes^43^– opening the way to the fabrication of a completely integrated micro total analysis system (μTAS) for bio applications. The hydro-dynamic focusing element was characterized by aligning incoming polystyrene (PS) microbeads with different sizes. To test the achievable focusing resolution, a proof of concept is then given, by using this element to optically detect the presence of bacteria in a water sample.

## Results & discussion

### Device design & simulation

**Figure 1:**
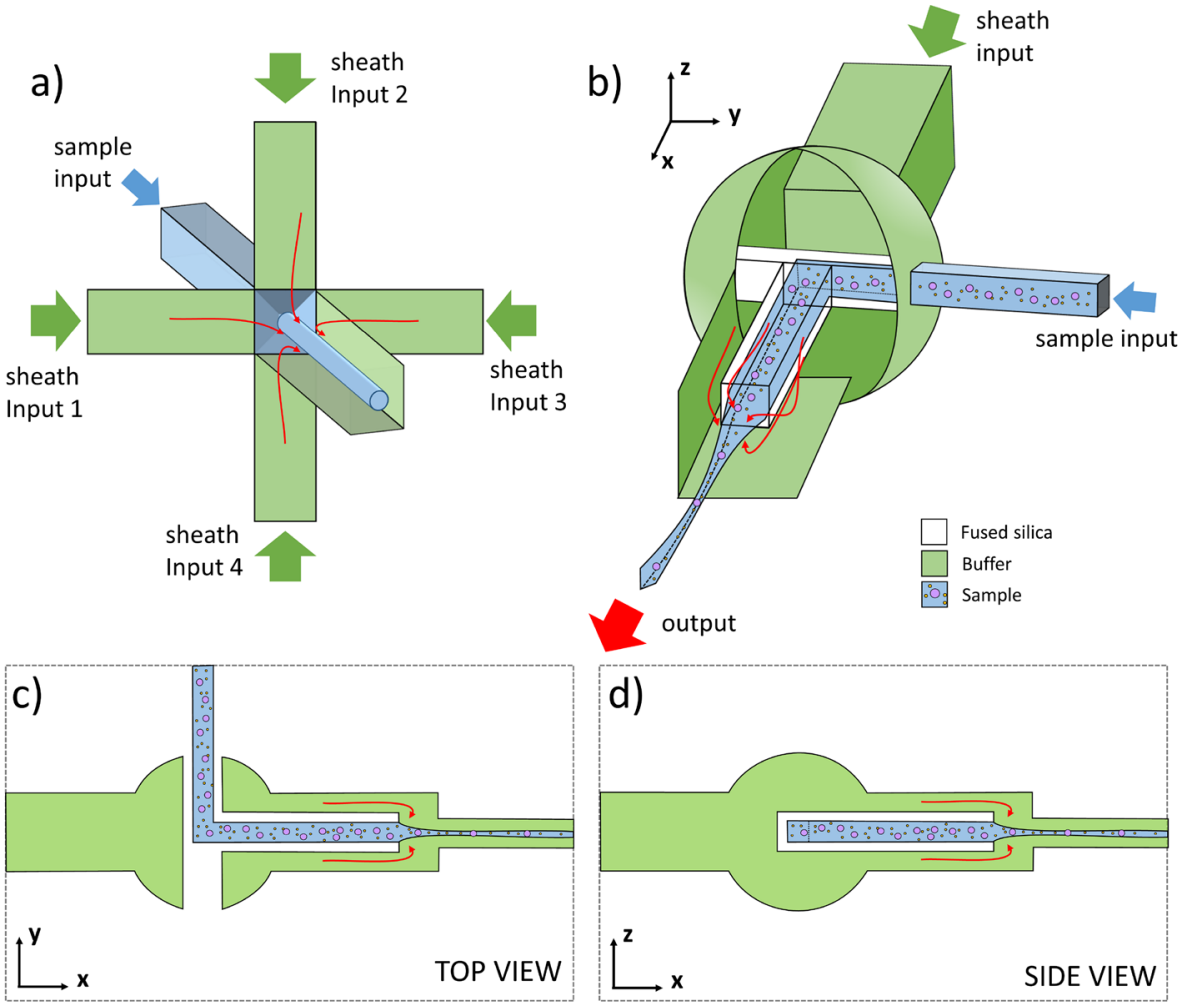
(**a**) The most used strategy to achieve a 3D hydrodynamic focusing is to confine the central flow within four sheath flows. Thus, the complexity of both the fabrication process and the operation of such devices greatly increases. (**b**) The designed geometry features only two inlet channels, one nested within the other. In this way, the buffer stream naturally confines the sample flow at the center of the outlet channel and with it all participle regardless of their size. (**c**) xy-plane view of the presented device. The sample channel is positioned in the middle of the sheath channel, so the outcoming flow is automatically aligned at the center of the outlet. This is valid for the zx-plane as well (**d**).

To achieve a precise 3D hydrodynamic focusing the presence of a confining sheath flow is necessary. Thus, due to the limitations of the current manufacturing techniques of microfluidic networks, the only way to shrink a central flow with a buffer fluid is to let them merge at a given point creating a multiple-branch junction (Fig. 1a). Therefore, both the fabrication process, that requires particular attention to not introduce asymmetries in the geometry, and the operation, that needs for the control of other four flows, are very complex. Indeed, any imbalance, due to the geometry, different flow viscosity or to the clogging of one of the branches, leads either to the focusing disruption or to a non-precise alignment. To avoid this, we designed a unique geometry that encloses a sample inlet within a buffering channel (Fig. 1b), in a non-deformable substrate (fused silica). By exploiting the capabilities of the FLICE fabrication technique, it was possible to design the 3D sample channel well align along the axis of the 3D sheath channel (Fig. 1c, d), ensuring accurate positioning of the focused flow in the centre of the outlet. In this way, thanks to laminarity, the sample flow is naturally confined by requiring a single buffer stream.

**Figure 2:**
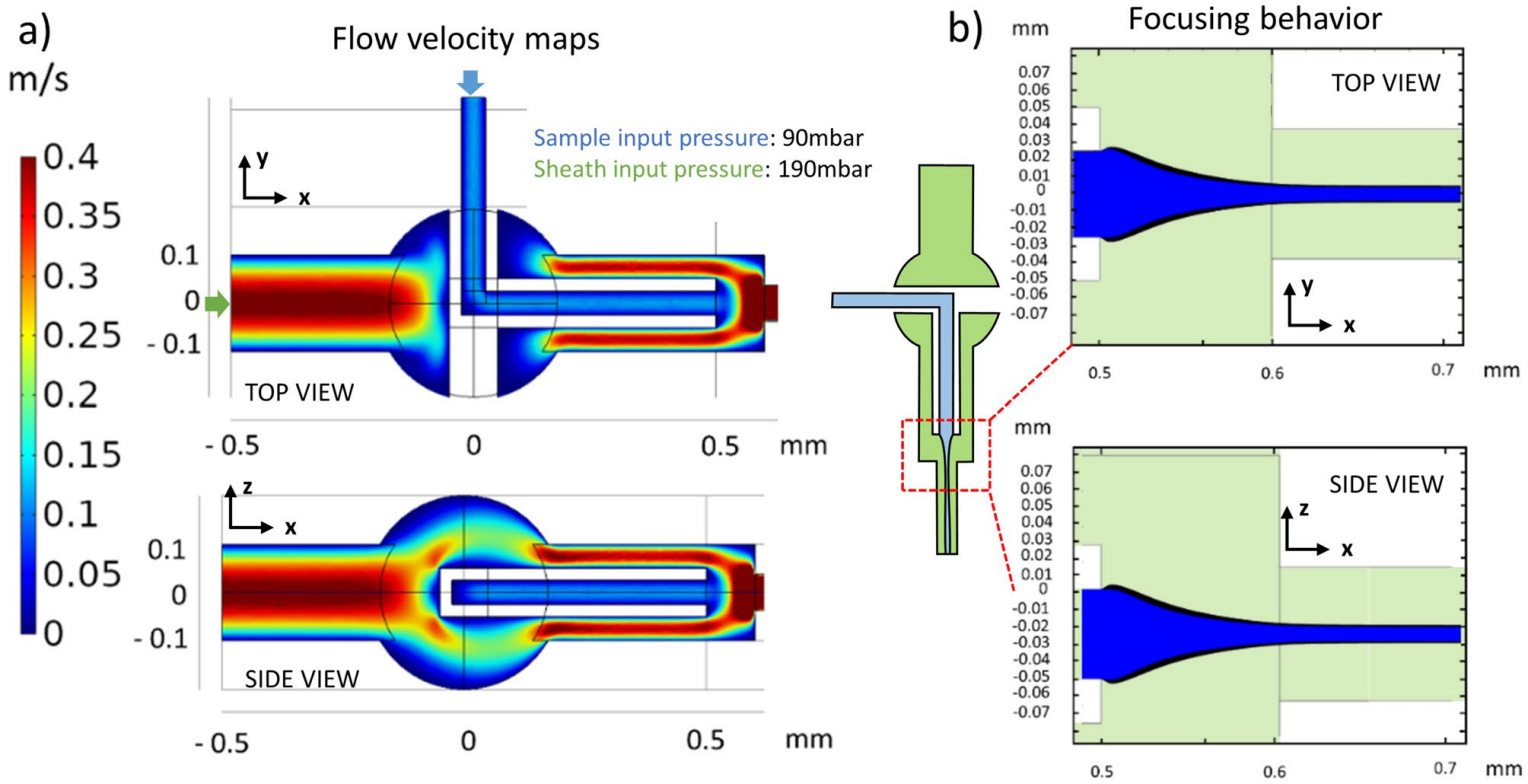
(**a**) Flow velocity map of the microfluidic channels in the xy and zx-plane, obtained with sample input pressures of 90 mbar and 190 mbar for the sheath inlet. The presence of the sample channel acts as an obstacle, but thanks to its “T” shape and the spherical expansion the downstream flow profile results in a uniform fluid velocity distribution both in the xy and zx plane. (**b**) The spherical enlargement also provides the device with a more powerful focusing behavior, resulting in a narrower focused stream.

Numerical simulations (COMSOL Multiphysics 5.3a) have been carried out to visualize the flow configuration of such a geometry and introduce some optimizations. For example, to ensure chip robustness and 3D symmetry of the various flows, the best results were obtained with the geometry shown in Fig. 2. The sample channel, with a sharp curve, is sustained to an un-etched fused silica support that contains it, modelled in the shape of a “T”. A spherical expansion of 200 μm radius was added to the junction of the two channels to ensure symmetry. In this way, the fluid can rearrange itself more uniformly around the “T” obstacle with a more uniform velocity distribution (Fig. 2a) and a narrower focused flow (Fig. 2b). For all simulations a hybrid solution for the mesh have been used: coarse mesh in the areas of least interest (e.g. the connection tubes), fine mesh for the 200 µm channel and super fine mesh for the entire sphere and the hydrodynamic focusing area.

Finally, simulations have also shown (Fig. 2b) how it is possible to further reduce the width of the confined stream by shrinking the dimensions of the outlet channel. Thus, the final geometry that has been fabricated is composed of all rectangular-section channels, where the buffer inlet has a 200 μm × 200 μm cross-section, the sample inlet 50 μm × 50 μm, and the outlet 120 μm ×120 μm. The total length of the channels is only 1.7 mm, resulting in a very compact device.

### Microfluidic results

**Figure 3:**
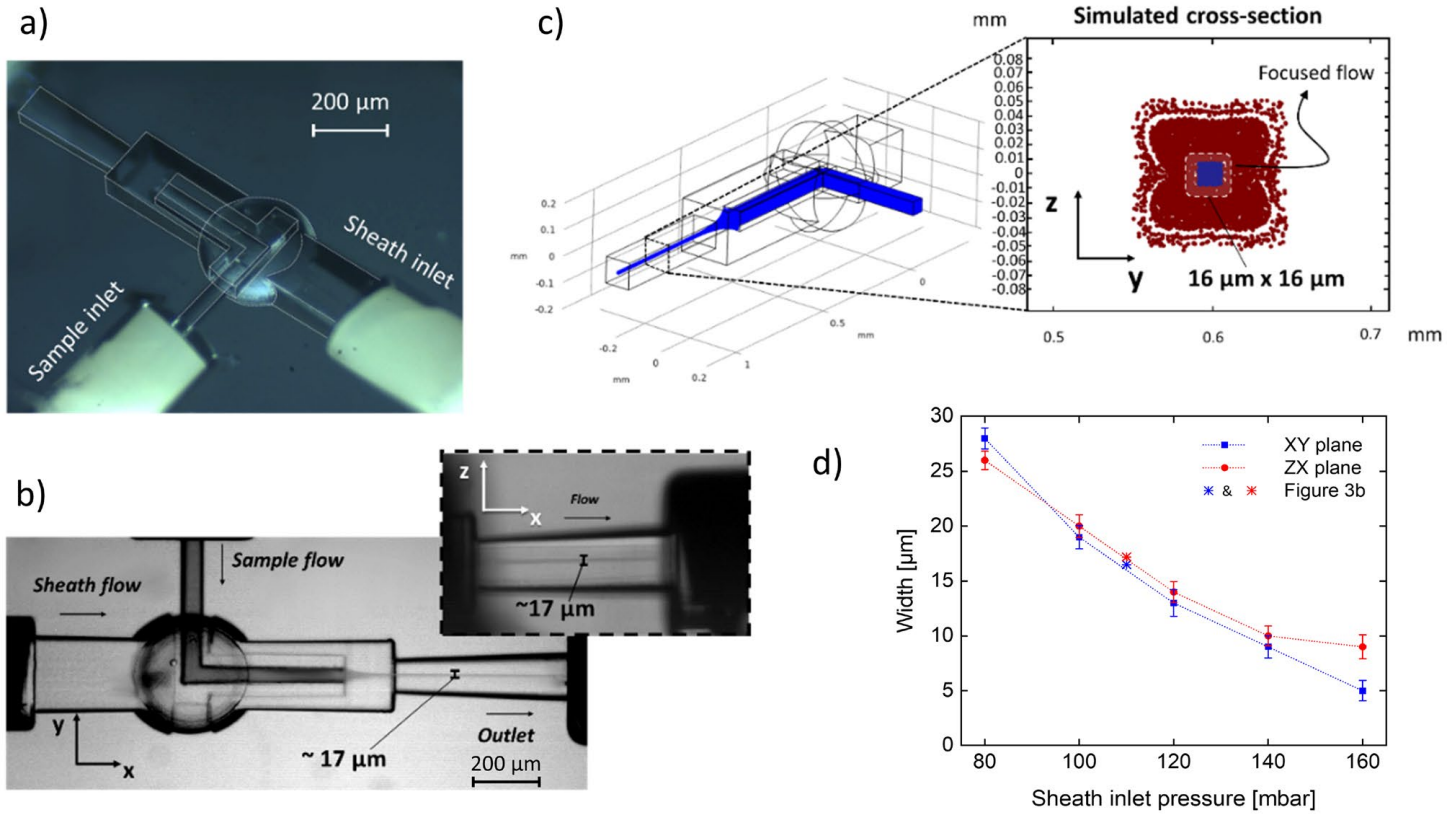
(**a**) Stereomicroscope image of the device with PEEK tubing inserted and ready to operate. (**b**) Optical microscope image (5 × magnification) of the device during the flow characterization experiments—sample input inlet pressure 80 mbar and sheath inlet pressure 110 mbar—. The channel widths were measured on another image (not shown) acquired with higher magnification objective (20×, NA 0.45, Resolution ~ 1 µm). (**c**) Simulated cross-section of the hydrodynamic focusing condition in the outlet (P_inlet sample_ = 80 mbar; P_inlet sheath_ = 110 mbar). The outermost red area (region of scattered red dots) is an artefact of the simulation due to the non-slip conditions of the walls, while the 16 μm × 16 μm blue area is the sample cross-section. (**d**) Experimental cross-section obtained in function of inlet sheath pressure. The inlet pressure has been set to 80 mbar, while the buffer inlet pressure has been scanned at 20 mbar steps starting from 60 to 160 mbar. The results with P_inlet sheath_ < 80 mbar cases are not shown, as no flow focusing has been observed due to the disadvantageous pressure couple. The confined stream shows good symmetry and the narrowest value reached is 9 μm × 5 μm, for the xy and the zx planes, respectively. As highlighted by the case study (Fig. 3b and points data with an asterisk), the experimental test validated the numerical simulations, resulting in a similar dimension and 3D symmetric focused flow.

The proposed geometry has the potential of achieving a full 3D hydrodynamic focusing within a single writing step, resulting in a very compact and easily operable device. Unlike other microfabrication technologies on soft materials, the capability and spatial resolution offered by the FLICE technique, allowed a very complex structure to be fabricated in a fast and robust way (Fig. 3a). The sample channel is suspended at the center of the buffering flow for almost 500 µm. We have not experienced any instability issues thanks to its 30 µm-thick walls directly connected to the bulk material. No sealing or annealing procedure is required to avoid mechanical weaknesses and fluid leakage. To visualize the focused flow, characterization tests have been carried out by injecting deionized water in the buffer channel and a blue-dyed water-based solution (Fig. 3b). The channel width was measured using a higher magnification objective (20×, NA 0.45) to ensure correct resolution (~ 1 µm).

Due to the extreme compactness of the device—with a total length 2 mm and a focusing section length of 0.62 mm—diffusion mechanisms have not been observed and the flow coming from the sample inlet has been correctly shrunk, both in the horizontal and in the vertical direction uniformly, matching the dimensions obtained by the numerical simulations (Fig. 3c, more details on this simulation are reported in Material and Method section). The relationship between the sample and the sheath input pressures has been investigated by choosing a reasonable pressure for the first (80 mbar), then varying the second with steps of 20 mbar (Fig. 3d). As expected, when the sheath flow is injected with a pressure lower than the sample inlet, no flow focusing is observable. Instead, when approaching the same value, a mild focusing effect starts to appear. This can be explained by the difference in the cross-section of the two channels (sample inlet: 50 µm × 50 µm, buffer inlet: 200 µm × 200 µm) that allows the sheath flow to reach higher velocities, besides the same injection pressure. Then, starting to create an imbalance between the two pressures, in favor of the sheath inlet, the sample flow is increasingly confined. Since the sample channel is centered along the sheath channel axis, the shrinkage is positioned in the middle of the cross-section of the outlet channel. By increasing the sheath pressure by more than twice the sample inlet, the focused stream disappears, since the buffer fluid creates virtual wall at the outlet of the sample channel, blocking the incoming flow. It can be noticed how the focused flow features a good symmetry, showing similar sizes both in the xy-plane and the zx-plane, except for the highest imbalance, where the dimension in the xy-plane is ~ 9 µm while it is 5 µm in the other plane. It should be appreciated how the device is capable to reach focusing values comparable with the literature, but with much less complexity in both in the fabrication process and in the operation (only two inlet channels needed). This suggests that the focusing potential of this geometry can even be pushed further—e.g., by reducing the diameter of the sample inlet.

**Figure 4:**
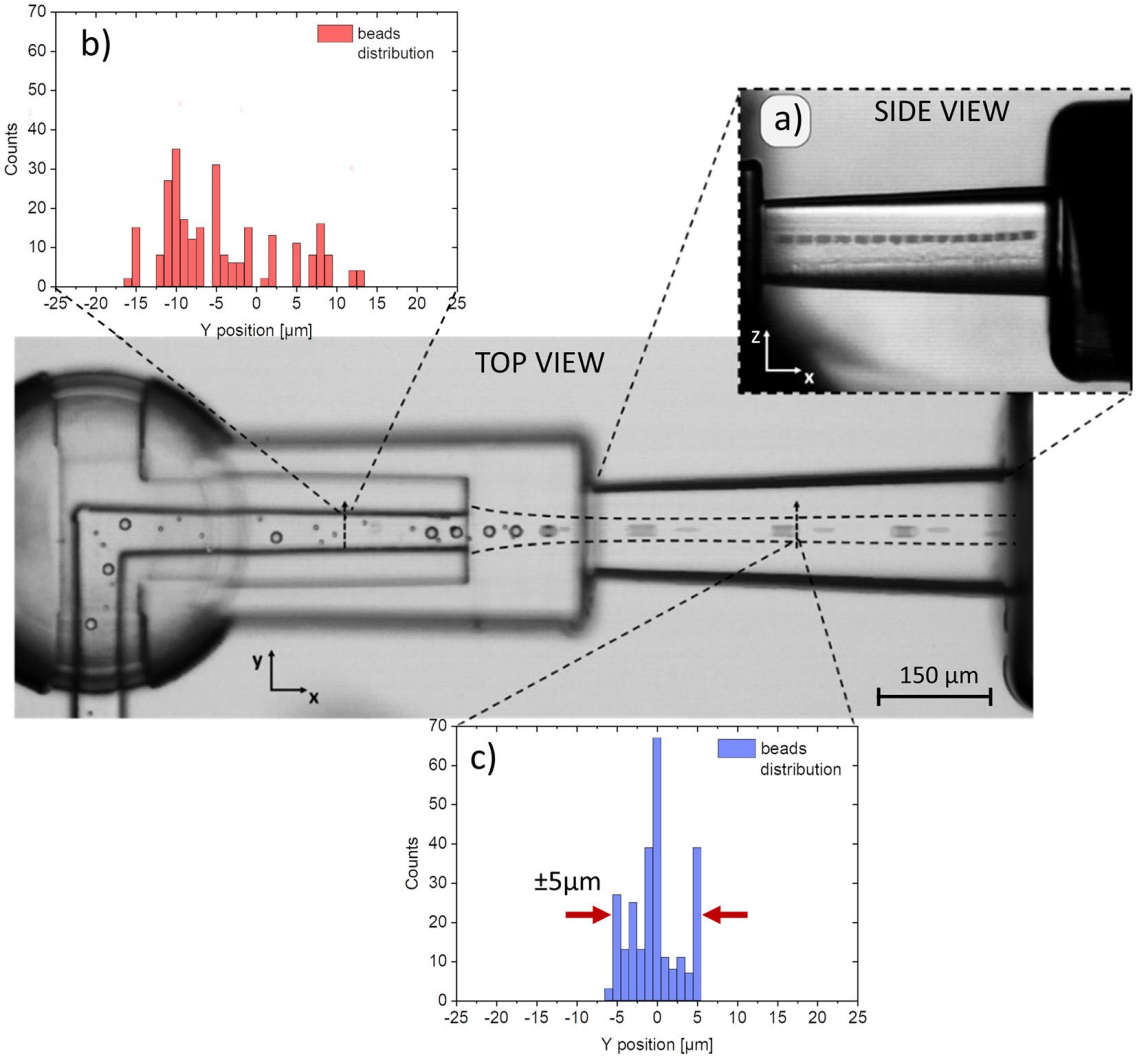
Proof of concept of the presented device. The microfluidic test has been carried out by injecting a water-based mixture of PS beads of different sizes—i.e., 15 μm and 6 μm. The image is a superimposition of multiple frames acquired at 10 000 fps through a high-speed camera, coupled to an optical microscope (10 × magnification). All the particles are correctly aligned along the outlet axis in 3D (inset **a**). The beads are randomly distributed across the sample channel cross section (inset **b**) but once they cross the focusing region, the redistribute in a narrower area (~ 10 μm) (Inset **c**). The count graphs are obtained by processing a whole video of about 1754 frames.

Since the target application of our device is the alignment of particles before crossing an interrogation area for single-particle analysis, PS beads focusing experiments have been also carried out. In an ideal application, such an element should present a good versatility, being able of centering particles of various sizes, without the need to modify either the flow or the geometry, unlike it happens in inertia-based techniques^18^. To verify this quality, we have tested our device by using a water-based mixture of particles—with diameters of 15 µm and 6 μm—and we have set the focusing dimension at larger particle size, keeping it constant. Figure 4 shows the obtained result: different particles are correctly focused along the central axis of the outlet channel, both in the xy-plane and in the zx-plane (Fig. 4, inset). As can be appreciated by the line profile analysis, the particles flowing in the sample channel are positioned randomly across the section but, after entering the focusing region, their distribution is centered around the outlet axis, showing a greater peak exactly in the middle of the channel. The ± 5 μm from this focused position is most likely because the confined flow has a dimension equal to larger particles. Thus, smaller particles are still able to occupy positions other than the exact center. However, transit areas of few microns are still compatible with most of interrogations methodologies.

**Figure 5:**
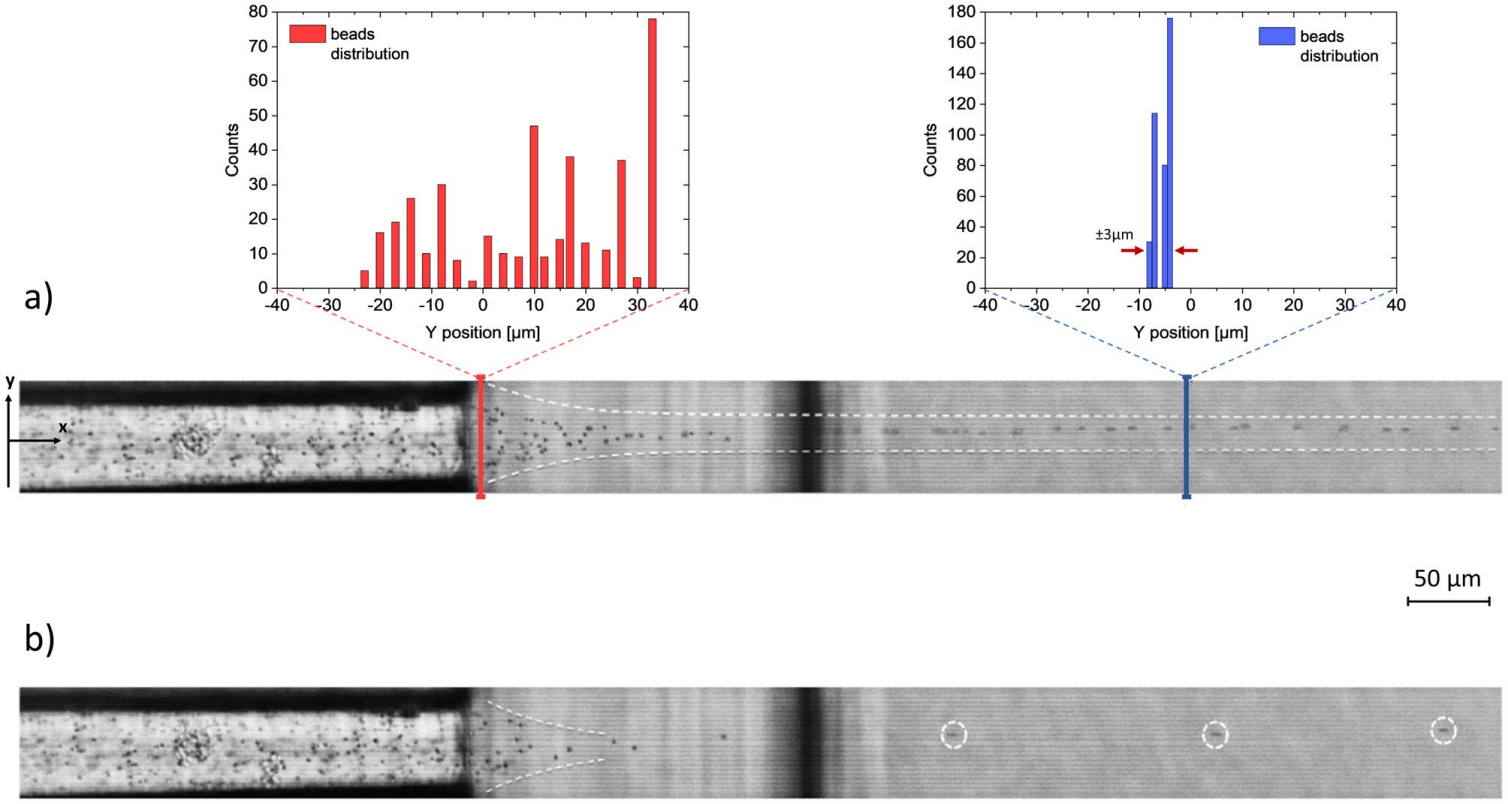
Microfluidic experiment of the same device by injecting a water-based solution of 1 μm PS beads (sample inlet pressure 97 mbar and sheath inlet pressure 194 mbar). (**a**) The image is an overlap of multiple frames acquired by a high-speed camera at 50 000 fps, coupled to an optical microscope (20 × magnification). The incoming particles are randomly distributed in the sample channel and then precisely aligned in the outlet channel. The alignment accuracy is extremely high, showing a possible displacement of only 3 μm. The line profile counting graphs are obtained by processing a video of about 2100 frames. (**b**) Single frame the acquired video, showing that particles, when undergoing the focusing region, are well divided and sent into the outlet channel one by one.

To better understand the potentialities of our device, not only in aligning, but also in separating clouds of incoming particles, we have pushed the particle sizes to the limit, by processing a water-based solution of 1 μm beads (Fig. 5). During the experimental phase, starting from the simulated inlet pressure conditions (sample 90 mbar, sheath 190 mbar), we further improved the focusing capability of the chip with respect to the small bead sample by slightly increasing the operating pressures (sample 97 mbar, sheath 194 mbar). Interestingly, the particles are again focused along the outlet axis, albeit the focused flow dimension is greater than 1 μm. The completely disorganized ensemble of 1 μm beads in the sample stream is sorted by the focusing region, resulting in a very well-peaked distribution in the outlet channel (Fig. 5a). Remarkably, regardless of how aggregated they may be, the particles, when undergoing the focusing flow, enter in the fluidic corridor one by one (Fig. 5b). In this case the confined positions differ by only 3 μm, showing an almost perfect alignment.

Moreover, from this experiment it was possible to validate the flow velocity map obtained by numerical simulations (Fig. 2 a). Thanks to the small size of the beads it is possible to assume that they move at the same speed as the flow. In this experiment, with sample inlet pressures of 97 mbar (simulated 90 mbar) and 194 mbar for the sheath inlet (simulated 190 mbar), particles were measured flowing at an average speed around 0.4 m/s similar to the flow in the simulation. Considering a cross-sectional transit area of ~ 15 μm × 15 μm for the sample flow (due to the focusing), the obtained actual throughput—i.e., volumetric consumption over time of just the sample to be processed—is 0.35 mL/h. However, the range of operating pressures can be much further extended, thanks to the solid mechanical properties of the device, reaching input values in the order of several bar. In this case, numerical simulations have shown that the focused flow speed approaches values in the order of tens of m/s, resulting in throughputs of tens of mL/h. Care should be taken when using fluids of different viscosities, as the working points (P_sample_ -P_buffer_) will be different from those typically shown here. However, it is still possible to identify working points that achieve the same focusing results.

**Figure 6:**
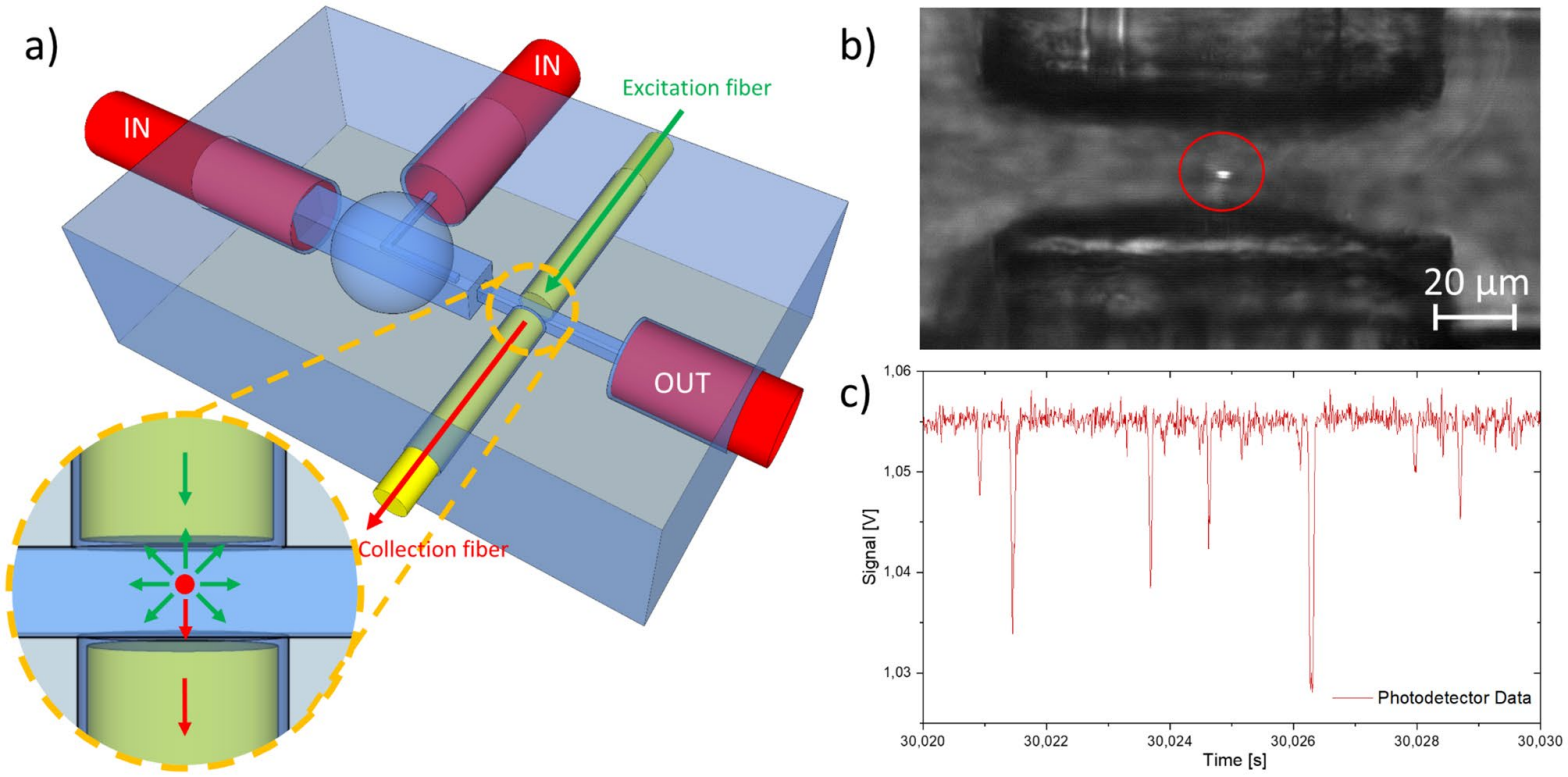
(**a**) Sketch of the optofluidic cytometer for single particle analysis. After the hydrodynamic focusing zone, the aligned particles pass through a couple of fibers (inset zoom). The scattering of the probe beam due to the particle allows his detection. (**b**) Single frame the acquired video, showing a bacteria that scatters light passing in the detection area (P_inlet sample_ = 97 mbar; P_inlet sheath_ = 194 mbar). (**c**) Typical signal acquired by the photodetector.

Finally, as proof of concept, the hydrodynamic focusing section has been integrated in a simple photo-cytometer chip for the particle's detection. Figure 6a shows the device sketch, the interrogation area is composed by two optical fibers (single mode fiber for the excitation branch and multimode for the collection one) placed one in front of the other. Thanks to this optical configuration the channel section (transverse to the flow) occupied by the laser beam is limited to a narrow line of approximately 5 µm in diameter, well located in the center of the main channel. When the particle passes in front of the fibers it obscures the probe beam, inducing a reduction of the light collected by the other fiber. The performance of the lab-on-a-chip for such automatic counting is closely related to the effective separation capability and accurate 3D position control of micrometric objects within channel. Displacements of a few microns relative to the optical axis of detection can inhibit the measurement, supporting the importance of the focusing concept developed previously. To test the achievable focusing resolution, a proof of concept, the validation of the chip was performed with Escherichia Coli bacteria in water solution. As shown in Fig. 6 b and c, when the bacteria is in the detection area it scatter enough light to be identified. This detection is label-free, and therefore not able to distinguish a bacteria from a particle with the same size. Nevertheless, by analyzing the peak width it is possible to recognize particles with different sizes.

## Conclusions

Microparticles analysis has recently aroused the attention of the microfluidics research field due to the wide range of information that can be extracted. In this context, particle manipulation within microfluidic networks becomes crucial. We successfully proposed, fabricated, and characterized a unique 3D flow focusing microfluidic device buried in fused silica substrate which brings together advantages such as compactness, high integrability, high throughputs, and ease-of-use operation. By designing a sample channel suspended inside a larger buffer channel, it has been possible to obtain a symmetric 3D hydrodynamic focusing less than 10 μm wide, by using just two inlets. This has allowed to confine in a central flow (spatially and temporally stable) a mixture of PS particles of 15 μm and 6 μm, with an accuracy of ± 5 μm and a solution of 1 μm PS beads, with an error of just 3 μm. FLICE manufacturing technique made it possible to carve the geometry from a single substrate of fused silica, providing the device with an exceptional mechanical robustness, and thus enabling high throughputs. In addition to its 3D manufacturing capabilities, this technique also offers the possibility of easily integrating self-aligned photonic elements to the microfluidic network-such as optical elements, optical fibers and waveguides-significantly simplifying the manufacturing steps.

All these unique advantages, combined with its extreme compactness (total length ~ 2 mm) and its totally clogging-free behavior, demonstrate that the proposed device exhibits all the features that could push forward the implementation of a totally microfluidic platform for biological and chemical particle sensing. Indeed the proposed geometry can be a fundamental tool for advanced studies on the highly sensitive detection of pollutants in fluids (bacteria, microplastics, heavy metals), for the analysis of cell stiffness and for the statistical study of all particles that can be made to emit (fluorescence) or scatter.

## Materials and methods

### Microfluidic simulations

Numerical simulations were performed using COMSOL Multiphysics 5.3a using the base and microfluidics modules. A hybrid mesh solution was used for all simulations: coarse mesh in the areas of least interest (e.g. the connection tubes), fine mesh for the 200 µm channel and super fine mesh for the whole sphere and the hydrodynamic focusing area. Boundary conditions were set to no-slip condition for the walls of the entire chip, while different pressures were applied to the inlets to simulate behavior under different circumstances. To correctly visualize (Fig. 3c) the cross section of two identical fluids (buffer and sample) under flow conditions, a suitable simulation strategy was used: the two flows (sample and buffer) were mimic as a set of small spheres of 50 nm (red for buffer molecules—blue for sample molecules) starting from their respective inlets and propagating through the device. For each inlet 10′000 beads were released, imposing the pressures reported in the capture (P_inlet sample_ = 80 mbar; P_inlet sheath_ = 110 mbar). The particles were set as not interacting with each other (bringing to excess a non-viscous fluid) while for the walls we impose non-slip conditions. Once stationary, the cross section was observed hundreds of microns away from the focusing zone.

### Device fabrication

FLICE technique^37–41^ is based on two main steps: femtosecond laser irradiation and wet etching. The first provides this manufacturing technique with an exceptional ability of to design geometries—even complex ones—in 3D. The process exploits the multi-photon absorption to selectively modify the material only in the spot volume, reaching spatial resolutions of less than 1 and 5 μm respectively in the transverse and longitudinal directions. Once exposed to the laser beam, the material is permanently modified and nano-structures—also called nanogratings—are created and aligned, depending on the light polarization^41^. By tuning it, it is possible to modulate the rate of the second step, a wet chemical etching, based on a water solution of hydrofluoric (HF) acid, with a 20% HF concentration. The chemical solution reacts faster with the modified material, allowing a selective removal that brings to the creation of hollow structures, starting from a single fused silica substrate^42–45^. Our micromachining setup consists of an amplified Yb:KGW femtosecond laser system (Pharos, Light Conversion) with 230-fs pulse duration, 515-nm wavelength (frequency doubled), 500-kHz repetition rate focused with a 50 × —0.42 NA microscope objective (M Plan Apo SL50X Ultra-Long Working Distance Plan-Apochromat, Mitutoyo). Computer-controlled, 3-axis motion stages (ABL-1000, Aerotech) interfaced by CAD-based software (ScaBase, Altechna) with an integrated acousto-optic modulator are used to translate the sample relative to the laser irradiation desiderate patch. Due to the accuracy needed to fabricate the internal channel, the parameters have been set to ensure a high rate etching step, exploiting the effect of different laser polarizations and a dense volume filling (with pitchs of 6 around 6 μm). The laser irradiation has been set to deliver an average power of 200 mW (400 nJ) on the sample, scanning it at a 1mm/s speed.

### Microfluidic experiments

The microfluidic experiments have been carried out throughout a controlled injection pressure system by Elveflow (OB1MK3), ensuring high resolution and stable pressure regulation. The fused silica geometry has been connected to the injection system by PEEK tubing of 150 μm of internal diameter and 7 cm length. To be able to observe fluids and particles flowing in the microfluidic device, the tests have taken place under an optical microscope (BX53M, Olympus) coupled to a high-speed camera (FASTCAM Mini UX100 type 800k-M-16G, Photron). Experiments have required the recording of videos at frame rates ranging from 10,000 up to 50,000 fps. Images have been acquired and processed through the Photron FASTCAM Viewer 4 (PVF4) software or ImageJ. To extract further information, on-purpose Matlab (MATLAB, version R201b, Natick, Massachusetts: The MathWorks Inc.) algorithm have been implemented and used.

The device has been designed to focus an incoming main flow and align particles carried within it along the central axis of the outlet channel. Several characterization experiments have been carried out. First, the flow configuration has been evaluated and compared to the results obtained by numerical simulations. To do so, a water-based blue dye solution has been injected in the sample inlet at a constant pressure of 80 mbar, while a deionized water solution has been flown in the sheath channel, varying its pressures from 60 to 160 mbar. Then, particle focusing behavior has been investigated, by using both a water-based mixture of 15 μm and 6 μm polystyrene beads (42716 & 42718 Polystyrene (PS) latex microsphere, 2.5 wt% dispersion, Alfa Aesar) and a solution of 1 μm particles (wt. 2.5%, Sigma-Aldrich), in two different experiments. Finally at the hydrodynamic focusing element has been add a couple of optical fibers (M42L01 and P1-460B-FC-1, Thorlabs) to detect the passage of particles in front of them. As proof of concept of biological application, this chip was tested for the detection of micro-organisms, in particular were used bacteria from the 25922 strain of Escherichia coli. Luria–Bertani (LB) broth and LB agar were used respectively for liquid culture and on-plate growth assays. The liquid bacterial cultures were grown overnight in LB medium in an incubator at a constant temperature of 37 °C, with a 200 rpm agitation rate. Before moving them to LB agar plates they were diluted to OD600 0.5. Then, for the in chip tests, they were suspended in water with random concentration. In terms of size, the bacteria used can be identified as rods of this nominal size: diameter 200–300 nm and length 1–2 µm.

## Data Availability

The datasets generated during and/or analysed during the current study are available from the corresponding author on reasonable request.
